# Malignancy Recorded Among Secondary Diagnoses and In-Hospital Mortality in Patients Hospitalized with Chronic Ulcers: A Nationwide Romanian Patient-Level Cohort Study

**DOI:** 10.3390/jcm15135261

**Published:** 2026-07-06

**Authors:** Mona Taroi (Yassin Cataniciu), Ilie Gligorea, Liliana Vecerzan (Novac), Doru Florian Cornel Moga, Sorin Radu Fleaca, Adrian Gheorghe Boicean, Cosmin Ioan Mohor, Adrian Nicolae Cristian, Horatiu Paul Domnariu, Carmen-Daniela Domnariu

**Affiliations:** 1 Faculty of Medicine, Lucian Blaga University of Sibiu, 550169 Sibiu, Romania; mona.yassincataniciu@ulbsibiu.ro (M.T.);; 2 Faculty of Military Management, Nicolae Bălcescu Land Forces Academy, 550170 Sibiu, Romania

**Keywords:** chronic ulcers, malignancy, in-hospital mortality, administrative data, ICD-10, patient-level analysis, wound healing

## Abstract

**Background/Objectives:** Chronic ulcers are common among older and multimorbid hospitalized patients and may reflect systemic vulnerability beyond the local wound condition. Malignancy recorded among secondary diagnoses may identify patients with reduced physiological reserve and increased inpatient risk, but its prognostic significance in hospitalized chronic ulcer populations remains insufficiently characterized. This study aimed to evaluate whether malignancy coded among secondary diagnoses was associated with in-hospital mortality among adults hospitalized with chronic ulcers. **Methods:** This nationwide retrospective cohort study used anonymized Romanian public-hospital discharge data for adults aged ≥18 years hospitalized with chronic ulcers between 1 January 2017 and 31 December 2022. The index-episode cohort included 69,349 patients generating 116,264 hospitalizations. Exposure was defined as at least one ICD-10 C00–C97 malignant neoplasm code recorded among secondary diagnoses in the relevant analytical hospitalization. The primary outcome was in-hospital mortality. Crude and adjusted odds ratios were estimated using logistic regression models. **Results:** Overall, 1837 patients had C00–C97 codes recorded among secondary diagnoses, with 73 deaths. In-hospital mortality was 3.97% among exposed patients and 1.78% among unexposed patients, corresponding to a crude odds ratio of 2.28 (95% CI 1.79–2.90). After adjustment for age group, sex, admission type, chronic ulcer category, and hospitalization pattern, malignancy recorded among secondary diagnoses remained associated with mortality (adjusted OR 1.87, 95% CI 1.42–2.45; *p* < 0.001). Additional adjustment for the number of non-malignant secondary diagnoses yielded similar results (adjusted OR 1.88, 95% CI 1.42–2.47; *p* < 0.001). **Conclusions:** Malignancy coded among secondary diagnoses may serve as a pragmatic administrative marker of increased in-hospital mortality risk among patients hospitalized with chronic ulcers. However, residual confounding and the absence of cancer-stage information limit causal interpretation.

## 1. Introduction

Chronic ulcers represent a major clinical and public health problem, particularly among older and multimorbid patients. Beyond their local cutaneous expression, chronic ulcers often reflect systemic vulnerability, impaired tissue repair, vascular and metabolic dysfunction, reduced mobility, and cumulative comorbidity burden. These wounds are frequently associated with prolonged care needs, recurrent hospitalizations, infection-related complications, and substantial healthcare resource use. Large-scale epidemiological studies have emphasized that chronic wounds impose a considerable burden on patients and healthcare systems, while also remaining difficult to quantify accurately because many cases are managed across multiple care settings and may be incompletely captured in routine data sources [1,2].

In Romania, the inpatient burden of chronic wounds has recently been quantified in our previous study, which provided national estimates of the prevalence, incidence, and in-hospital mortality of hospitalized patients with chronic wounds. These estimates were generated using a large-scale data analytics approach applied to National Inpatient Database records from public hospitals between 2017 and 2022. That study demonstrated the feasibility of using administrative hospital data for population-level wound research and highlighted in-hospital mortality as an indicator of severe clinical burden in this population [3].

In-hospital mortality among patients with chronic ulcers should not be attributed solely to the wound itself. It is more likely to capture the combined effect of ulcer phenotype, multimorbidity, acute illness, and reduced physiological reserve. Patients admitted with chronic ulcers often have advanced age, cardiovascular disease, diabetes, renal impairment, infection, immobility, or malnutrition, all of which may contribute to poor inpatient outcomes [4,5]. Therefore, in-hospital death may identify a clinically vulnerable subgroup in whom chronic ulceration coexists with severe systemic disease.

Malignancy may represent one such systemic factor. Cancer can influence prognosis through systemic inflammation, cachexia, immune dysfunction, nutritional deterioration, increased susceptibility to infection, treatment-related toxicity, and reduced functional reserve. Cancer cachexia is associated with skeletal muscle wasting, metabolic disturbance, impaired tolerance to treatment, poorer quality of life, and reduced survival. These mechanisms are also relevant to wound healing. Adequate tissue repair requires preserved immune function, nutritional substrates, vascular supply, and sufficient systemic resilience. Therefore, cachexia and chronic inflammatory states may contribute to delayed healing, increased complication risk, and poorer outcomes in patients with chronic wounds [6,7].

Despite this plausible clinical link, the prognostic role of malignancy recorded among secondary diagnoses (hereafter, coded malignancy) in patients hospitalized with chronic ulcers remains insufficiently characterized at population level. Previous work has shown that patients with chronic ulcers have increased long-term mortality and that mortality risk may vary according to ulcer etiology, supporting the need for better prognostic characterization in wound-care populations [8]. In parallel, oncology-focused literature indicates that wound healing in patients with cancer may be affected by the oncological disease process itself, nutritional deterioration, radiotherapy, chemotherapy, and other treatment-related factors [9,10]. However, most chronic ulcer studies focus on diabetes, peripheral arterial disease, venous disease, infection, amputation, recurrence, or wound-care costs, while coded malignancy is rarely examined as a distinct secondary diagnosis associated with in-hospital death. Similarly, oncology-related wound-healing studies often address surgical wounds, radiotherapy-related tissue injury, chemotherapy-related impairment, or cancer-specific wound complications rather than broad populations of patients hospitalized for chronic ulcer disease. This gap is particularly relevant because individualized risk prediction and risk stratification remain important challenges in chronic wound management [11].

Administrative hospital databases offer an opportunity to explore this gap at scale. Although such data cannot provide detailed information on cancer stage, disease activity, treatment, performance status, or wound severity, they allow patient-level identification of malignancy codes recorded among secondary diagnoses and their association with hard outcomes such as in-hospital death. This approach may help determine whether malignancy recorded among secondary diagnoses functions as an administrative marker of acute systemic severity among hospitalized chronic ulcer patients.

Therefore, the aim of this study was to evaluate whether coded malignancy, defined as ICD-10 C00–C97 codes recorded among secondary diagnoses [12], was associated with in-hospital mortality among patients hospitalized with chronic ulcers, using a nationwide patient-level analysis. The primary analysis was conducted in the overall cohort. Single-admission and recurrent patients were further examined in secondary exploratory stratified analyses, recognizing that hospitalization pattern may be influenced by survival time.

## 2. Materials and Methods

### 2.1. Study Design, Data Source, and Population

This nationwide retrospective cohort study used administrative hospitalization data from Romanian public hospitals to examine the association between malignancy coded among secondary diagnoses and in-hospital mortality in adults hospitalized with chronic ulcers. The source dataset was obtained from the National Institute of Public Health, Bucharest, Romania, after a formal request for anonymized records of patients aged ≥18 years who were discharged between 1 January 2017 and 31 December 2022 with ICD-10 codes consistent with chronic ulcer disease.

Hospitalizations were identified using ten predefined ICD-10 chronic ulcer codes. For each episode, the database included an anonymized patient identifier, age at admission, sex, area of residence, socio-professional status, admission date, length of hospital stay, type of admission, hospital department, principal diagnosis at discharge, secondary diagnoses, and discharge status. Discharge status was used to identify in-hospital death.

The final dataset included 116,264 hospitalizations generated by 69,349 adult patients hospitalized with chronic ulcers during the study period. Of these patients, 50,493 had a single hospitalization and 18,856 had multiple hospitalizations. The same national administrative data source and analytical framework were previously used to estimate the prevalence, incidence, and in-hospital mortality of hospitalized chronic ulcers in Romania.

**Figure 1:**
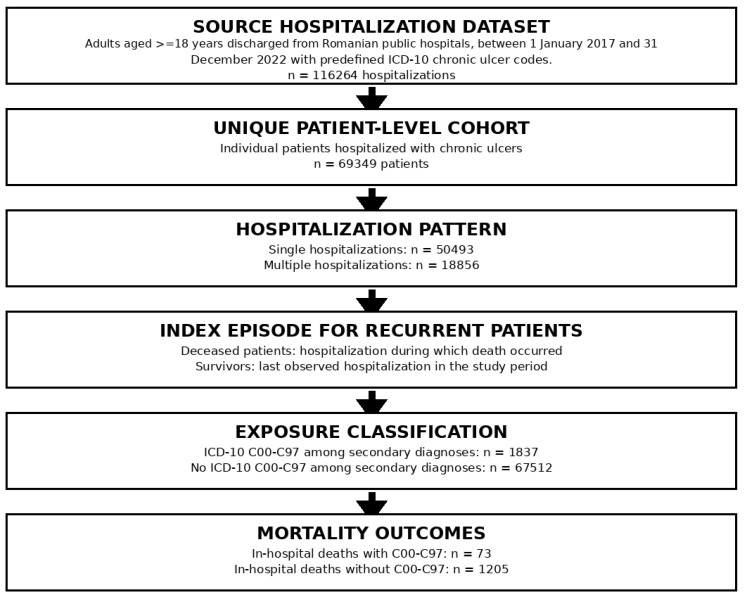
Patient selection and analytical cohort construction. Note: ICD-10 codes C00-dashC97 refer to malignant neoplasms.

The construction of the patient-level index-episode cohort, including hospitalization-pattern stratification, exposure classification, and in-hospital death counts, is summarized in Figure 1.

Chronic ulcer hospitalizations were identified using ICD-10 codes recorded as principal discharge diagnoses and grouped into six chronic ulcer categories: venous ulcers (I83.x), arterial ulcers (I70.23), diabetic ulcers (E1x.73), pressure ulcers (L89), non-classified lower-limb ulcers (L97), and chronic skin ulcers not elsewhere classified (L98.4). Age was analyzed as mean and standard deviation and using predefined categories: <45 years, 45–54 years, 55–64 years, 65–74 years, 75–84 years, and ≥85 years.

### 2.2. Patient-Level Index-Episode Construction

Although the source dataset contained hospitalization records, the analysis was performed at patient level to avoid over-representation of individuals with repeated admissions. For patients with a single admission, the analytical episode was the only available record.

For recurrent patients, one index episode was selected for mortality analysis. Among those who died in hospital, the index episode was the admission in which death was recorded. Among survivors, it was the last available admission during the study period. Secondary diagnoses from the analytical episode were used to define malignancy status and index-episode covariates.

This strategy was chosen to preserve a clinically interpretable patient-level analysis while avoiding repeated contributions from the same patient. The death admission was selected for deceased recurrent patients because it represented the terminal inpatient episode in which the primary outcome occurred. For recurrent survivors, the last observed admission provided the most recent available secondary diagnosis profile. However, this approach may introduce survivor-related or time-dependent bias, because recurrent patients must survive long enough to accumulate repeated admissions, whereas patients who die early cannot subsequently contribute to a recurrent trajectory. Therefore, analyses stratified by hospitalization pattern were considered secondary and exploratory and were interpreted cautiously.

This approach allowed each patient to contribute once to the main analysis while preserving a clinically relevant episode for comparison between deceased and surviving patients.

### 2.3. Exposure, Outcome, and Covariates

The primary exposure was malignancy recorded among secondary diagnoses. Patients were classified as exposed if they had at least one ICD-10 C00–C97 malignant neoplasm code recorded among secondary diagnoses in the analytical hospitalization. This definition included primary malignant tumors, metastatic malignant neoplasms, and hematologic malignancies coded as secondary diagnoses. However, because the analysis was based on administrative discharge data, C00–C97 codes recorded among secondary diagnoses did not necessarily distinguish active cancer, treated cancer, cancer in remission, historical cancer, metastatic disease not explicitly coded as such, or treatment-related oncological status. Therefore, the exposure should be interpreted as administratively coded malignancy during the analytical hospitalization rather than as clinically verified active cancer. In situ neoplasms, benign neoplasms, and neoplasms of uncertain or unknown behavior (ICD-10 D00–D49) were not included in the primary exposure definition.

The primary outcome was in-hospital mortality, defined as death recorded in the discharge status field. Patients without a recorded in-hospital death were classified as survivors.

Covariates were selected according to clinical relevance and availability in the administrative database. The adjustment variables included age group, sex, type of admission, chronic ulcer category, and hospitalization pattern. Type of admission was recorded as emergency versus non-emergency/other, with all non-emergency admission categories grouped together. A sensitivity model additionally adjusted for the number of non-malignant secondary diagnoses recorded during the analytical hospitalization. This variable was used as a pragmatic administrative proxy for overall coded comorbidity burden, but it was not considered equivalent to a validated comorbidity index. A Charlson or Elixhauser comorbidity index was not constructed because the available administrative dataset did not permit reliable implementation of a validated comorbidity algorithm for this analysis. In particular, the cancer and metastatic cancer components of these indices overlap directly with the primary exposure definition based on ICD-10 C00–C97 codes, which could have introduced circular adjustment or overadjustment. In addition, the database did not include the clinical detail needed to distinguish chronic comorbidities from acute complications, disease severity, frailty, nutritional status, or functional impairment. Because diagnosis counts in administrative data may reflect both true morbidity burden and coding intensity or documentation practices, this adjustment was interpreted as a sensitivity analysis. It was not considered complete control for comorbidity.

### 2.4. Statistical and Sensitivity Analyses

Descriptive analyses compared patients with and without malignancy recorded among secondary diagnoses. Categorical variables were summarized as absolute numbers and percentages, while age was reported as mean and standard deviation and by predefined age group. Group comparisons used χ^2^ tests [13]; Fisher’s exact test was additionally reported for 2 × 2 analyses with small cell counts [14]. Continuous variables were compared using the *t*-test or Mann–Whitney U test, depending on distributional assumptions [15].

The crude association between malignancy recorded among secondary diagnoses and in-hospital mortality was estimated using odds ratios (ORs) with 95% confidence intervals (CIs) [16]. Adjusted associations were evaluated using logistic regression models. Model 1 included age group, sex, and type of admission. Model 2 additionally included the chronic ulcer category and hospitalization pattern in the overall cohort. In stratified analyses, hospitalization pattern was not included because patients were already analyzed separately as single-admission or recurrent patients. Model 3 was a sensitivity model that further adjusted for the number of non-malignant secondary diagnoses recorded during the analytical hospitalization, used as a pragmatic administrative proxy for coded comorbidity burden.

Because the logistic regression models were used primarily for association estimation rather than clinical prediction, model robustness was assessed mainly by comparing the malignancy estimates across adjustment levels and sensitivity analyses. To improve transparency, descriptive model fit and performance metrics were also calculated for the adjusted models, including the area under the receiver operating characteristic curve (AUROC/c-statistic), Akaike information criterion (AIC), McFadden pseudo-R^2^, and Brier score [17]. These metrics were interpreted as apparent in-sample diagnostics and not as externally validated predictive performance measures.

The primary analysis was conducted in the overall cohort of 69,349 patients. Stratified analyses among single-admission and recurrent patients were considered secondary and exploratory. Because only eight deaths occurred among recurrent patients with malignancy recorded among secondary diagnoses, Firth penalized logistic regression was fitted as a rare-event sensitivity analysis for this subgroup [18,19]. Recurrent-patient estimates were therefore interpreted cautiously.

Additional supportive analyses were performed to assess the internal consistency and clinical heterogeneity of malignancy coding. First, we examined whether patients with ICD-10 C00–C97 codes also had additional secondary diagnoses compatible with oncological burden or cancer-related care, including metastatic or unspecified malignant neoplasm codes (C77–C80), cachexia (R64), malnutrition (E43–E46), anemia in neoplastic disease (D63.0), radiotherapy or chemotherapy session codes (Z51.0–Z51.1), and personal history of malignant neoplasm (Z85.x). This analysis was supportive and did not represent clinical validation against a cancer registry.

Second, malignant codes were grouped into five non-mutually exclusive binary categories: metastatic/secondary malignant neoplasms (C77–C79), malignant neoplasm without specified site (C80), solid non-skin malignancies (C00–C76 excluding C43–C44), hematologic malignancies (C81–C96), and skin malignancies (C43–C44). Solid non-skin malignancies were further described in nine site-based subcategories in the Supplementary Material. Because these categories were non-mutually exclusive, patients could contribute to more than one category when multiple malignant codes were recorded during the analytical hospitalization.

Individual malignant ICD-10 codes were assessed in relation to in-hospital mortality as exploratory, hypothesis-generating analyses only. Because code-level analyses involved sparse cell counts and multiple testing, these findings were not used for the primary interpretation.

To assess the robustness of the findings to potential bias introduced by index-episode selection, results were also examined separately among single-admission patients, for whom no index-episode selection was required. This subgroup was considered less susceptible to selection bias related to choosing an analytical episode. Recurrent-patient results were reported separately and interpreted as secondary and exploratory because the selected episode may depend on survival time and hospitalization history.

No correction for multiple testing was applied to the exploratory malignancy-category or individual ICD-10 code-level analyses because these analyses were not intended for confirmatory inference. They were performed only to describe potential coding patterns and to generate hypotheses for future studies; therefore, the corresponding *p*-values were interpreted descriptively.

Analyses were performed using Python 3.13.5 with pandas 2.2.3, SciPy 1.17.0, and statsmodels 0.14.6. Before fitting the regression models, missingness was assessed for all variables included in the adjusted analyses, including in-hospital mortality, malignancy status, age group, sex, admission type, chronic ulcer category, hospitalization pattern, and the number of non-malignant secondary diagnoses. No missing values were identified for these variables in the index-episode analytical cohort; therefore, complete-case analysis did not exclude any patients from the regression models. The number of patients included in each model is reported. Statistical significance was defined as *p* < 0.05. Reporting was aligned with STROBE and RECORD principles for observational studies using routinely collected administrative data [20,21]. Data quality checks, model specifications, the distribution of non-malignant secondary diagnosis count, rare-event sensitivity analyses, and exploratory code-level analyses are provided in the Supplementary Materials.

### 2.5. Ethical Considerations

This study used an anonymized administrative database in which all personal identifiers were encoded before delivery to the authors. Access to the data was granted under a data-sharing agreement with the National Institute of Public Health, in compliance with the EU General Data Protection Regulation (GDPR) and national legislation on the secondary use of health data. The study protocol was approved by the Scientific Research Ethics Committee of “Lucian Blaga” University of Sibiu (approval No. 11/14 March 2025).

## 3. Results

### 3.1. Study Cohort and Baseline Characteristics

The final study cohort included 69,349 adult patients hospitalized with chronic ulcers between 1 January 2017 and 31 December 2022, generating 116,264 hospitalizations. Of these patients, 50,493 had a single hospitalization and 18,856 had multiple hospitalizations during the study period.

Using the index-episode definition of exposure, 1837 patients had at least one ICD-10 C00–C97 malignancy code recorded among secondary diagnoses in the relevant analytical hospitalization. This included 1392 patients with a single hospitalization and 445 patients with multiple hospitalizations. In the overall cohort, 73 deaths occurred among patients with C00–C97 codes, corresponding to an in-hospital mortality of 3.97%, compared with 1.78% among patients without C00–C97 codes. These crude mortality differences are further examined in the primary association analyses below.

Baseline characteristics according to malignancy status are presented in Table 1. Compared with unexposed patients, those with C00–C97 codes were older, both by mean age and age-group distribution, with higher proportions in the 65–74, 75–84, and ≥85-year categories. Sex distribution was similar between groups. Differences were also observed in residence, ulcer category, and hospitalization pattern. The exposed group included a higher proportion of urban residents, more patients with chronic skin ulcers not elsewhere classified and pressure ulcers, and fewer patients with venous ulcers. Patients with C00–C97 codes were also slightly more often single-admission patients. In-hospital death was more frequent in the exposed group; this crude mortality difference is analyzed further in Table 2 and Table 3.

### 3.2. Primary Association Between Malignancy and In-Hospital Mortality

The crude association between malignancy recorded among secondary diagnoses and in-hospital mortality is shown in Table 2. In the overall cohort, patients with C00–C97 codes had higher in-hospital mortality than those without such codes: 3.97% versus 1.78%, corresponding to a crude OR of 2.28 (95% CI 1.79–2.90; *p* < 0.001). A similar pattern was observed among single-admission patients, whereas the association among recurrent patients was imprecise and not statistically significant.

Adjusted logistic regression results are presented in Table 3. Because no missing values were identified for the variables included in the models, all eligible patients in each analytical cohort contributed to the corresponding analyses. In the overall cohort, malignancy recorded among secondary diagnoses was associated with higher odds of in-hospital death after adjustment. The age-, sex-, and admission type-adjusted estimate in Model 1 was 2.42 (95% CI 1.88–3.10; *p* < 0.001). After additional adjustment for chronic ulcer category and hospitalization pattern in Model 2, the OR decreased to 1.87 (95% CI 1.42–2.45; *p* < 0.001), corresponding to 87% higher adjusted odds. In the sensitivity Model 3, further adjustment for the number of non-malignant secondary diagnoses produced an almost identical estimate (OR 1.88, 95% CI 1.42–2.47; *p* < 0.001). Additional stratified Model 3 estimates for single-admission and recurrent/index-episode patients are reported in Supplementary Table S1.

In stratified analyses, the association was also observed among single-admission patients in Model 2 (OR 1.93, 95% CI 1.44–2.58; *p* < 0.001), whereas recurrent/index-episode estimates were above 1 but statistically imprecise and crossed the null, including in the Firth penalized sensitivity model.

Descriptive model fit and apparent in-sample performance metrics are reported in Supplementary Table S1. These metrics are provided for transparency and should be interpreted descriptively, as the regression models were designed primarily for association estimation rather than clinical prediction.

The similarity between Model 2 and the sensitivity Model 3 suggests that the association between C00–C97 codes and in-hospital mortality was not materially altered after adjustment for the overall number of coded non-malignant secondary diagnoses. However, this variable should be interpreted as a pragmatic administrative proxy rather than as a validated or complete measure of comorbidity burden.

**Figure 2:**
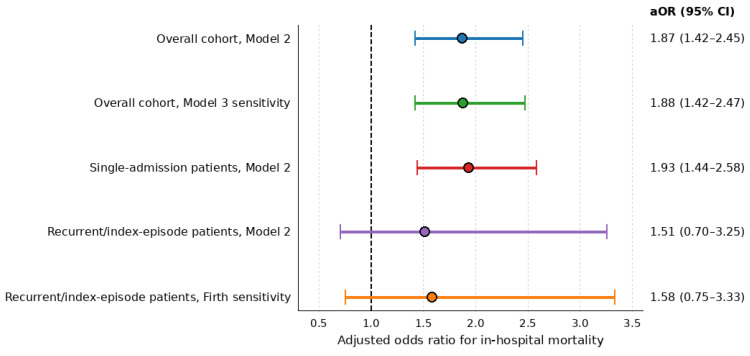
Adjusted odds ratios for in-hospital mortality associated with malignancy recorded among secondary diagnoses. Abbreviations: OR, odds ratio; CI, confidence interval. The dashed vertical line indicates the null value (OR = 1); colored horizontal lines indicate 95% confidence intervals for the corresponding models/subgroups.

The adjusted estimates from the main and sensitivity models are summarized in Figure 2. The association remained significant in the overall cohort and among single-admission patients, while recurrent-patient estimates were imprecise, crossed the null value, and should not be interpreted as evidence of absence of association because only eight exposed deaths occurred in this subgroup. Detailed sensitivity analyses, including the distribution of the non-malignant secondary diagnosis count and the Firth penalized recurrent-subgroup models, are provided in Supplementary Table S1.

Model 2 was adjusted for age group, sex, type of admission, chronic ulcer category, and hospitalization pattern in the overall cohort. In stratified analyses, hospitalization pattern was not included because patients were already analyzed separately as single-admission or recurrent/index-episode patients. Model 3 was a sensitivity model additionally adjusted for the number of non-malignant secondary diagnoses recorded during the analytical hospitalization. Firth sensitivity refers to Firth penalized logistic regression fitted in the recurrent/index-episode subgroup because of the small number of exposed deaths. Estimates for the recurrent/index-episode subgroup were based on only eight exposed deaths and should be interpreted as exploratory and statistically imprecise.

### 3.3. Exploratory Stratified Analyses by Hospitalization Pattern

Stratified analyses by hospitalization pattern were conducted as secondary exploratory analyses. Because hospitalization pattern is influenced by survival time, these comparisons may be affected by survivor-related or time-dependent bias and should not be interpreted as causal differences between single-admission and recurrent patients.

Among patients with a single hospitalization, 1392 had C00–C97 codes and 65 deaths occurred in this group. In-hospital mortality was 4.67% among single-admission patients with malignancy codes, compared with 1.98% among those without such codes, corresponding to a crude OR of 2.43 (95% CI 1.88–3.14; *p* < 0.001). The association persisted after adjustment in Model 2, with an OR of 1.93 (95% CI 1.44–2.58; *p* < 0.001). The persistence of the association among single-admission patients was considered an important sensitivity finding, because this subgroup did not require index-episode selection and was therefore not affected by bias related to choosing an analytical episode among recurrent patients.

Among recurrent patients, 445 had C00–C97 codes in the index episode and eight deaths occurred in this group. In-hospital mortality was 1.80% among recurrent patients with malignancy codes and 1.28% among those without such codes, corresponding to a crude OR of 1.42 (95% CI 0.70–2.88; *p* = 0.335). Adjusted point estimates remained above 1, but the confidence intervals were wide and crossed the null value, indicating statistical imprecision. The Model 2 OR was 1.51 (95% CI 0.70–3.25; *p* = 0.293), and the Firth penalized sensitivity model yielded a similar estimate (OR 1.58, 95% CI 0.75–3.33; *p* = 0.226). Because only eight deaths occurred among exposed recurrent patients, this subgroup analysis was considered exploratory and underpowered; therefore, the lack of statistical significance should not be interpreted as evidence of absence of association.

### 3.4. Mortality by Ulcer Category Among Patients with Coded Malignancy

Among patients with C00–C97 codes recorded among secondary diagnoses, in-hospital mortality varied by chronic ulcer category. Mortality was highest among patients with pressure ulcers and arterial ulcers. In the overall cohort, mortality was 24.22% among patients with pressure ulcers and 7.69% among those with arterial ulcers. A similar pattern was observed among single-admission patients, with mortality of 24.49% in pressure ulcers and 8.15% in arterial ulcers.

These ulcer-category-specific mortality percentages were descriptive only and were calculated within each chronic ulcer category among patients with malignancy codes. They should not be interpreted as adjusted comparisons between ulcer categories or as evidence of an independent effect of ulcer category on in-hospital mortality. No formal interaction analysis between malignancy status and chronic ulcer category was performed. Detailed ulcer-category mortality results are provided in Supplementary Table S5.

### 3.5. Exploratory Analyses of Malignancy Categories and Individual ICD-10 Codes

To explore whether the overall C00–C97 association was driven by specific malignancy patterns, secondary analyses were performed by malignancy category and by individual malignant ICD-10 code. In non-mutually exclusive analyses of C00–C97 categories, metastatic or secondary malignant neoplasm codes (C77–C79), malignant neoplasm without specified site (C80), and solid non-skin malignancies showed the strongest crude associations with in-hospital mortality. Hematologic malignancies had a higher point estimate but did not reach statistical significance. Skin malignancy codes (C43–C44) showed low observed in-hospital mortality; however, this finding was not interpreted as protective because the category is clinically heterogeneous and includes many non-melanoma skin cancers. Among solid non-skin malignancy subcategories, most site groups showed significant crude associations with mortality, except head and neck and breast malignancies. These analyses were exploratory, non-mutually exclusive, and are reported in Supplementary Table S2.

Individual malignant ICD-10 codes were also explored in relation to in-hospital mortality. The complete code-level results are provided in Supplementary Table S3. Because these analyses involved sparse event counts and multiple comparisons, they were considered exploratory and hypothesis-generating only. Accordingly, individual ICD-10 code-level findings were interpreted descriptively and were not used for the primary interpretation of the study findings. Consistent with the exploratory purpose of these analyses, no correction for multiple testing was applied, and the resulting *p*-values should be interpreted descriptively rather than as confirmatory evidence.

### 3.6. Supportive Internal Consistency Analysis

In a supportive internal consistency analysis, 308 of 1837 patients with C00–C97 codes (16.77%) had at least one strict supportive oncological pattern, including C77–C80, R64, E43–E46, D63.0, Z51.0–Z51.1, or Z85.x. Metastatic, secondary, or unspecified malignant neoplasm codes (C77–C80) were present in 202 exposed patients (11.00%). Non-C supportive codes were substantially more frequent among patients with C00–C97 than among those without C00–C97, including cachexia, malnutrition, anemia in neoplastic disease, oncology-related care codes, and personal history of malignancy. These findings supported the internal consistency of malignancy coding in the administrative database but did not constitute clinical validation against a cancer registry. The complete results are provided in Supplementary Table S4. These supportive codes were used only to assess internal coding consistency and were not used to reclassify patients as having active, historical, metastatic, or treated cancer.

## 4. Discussion

### 4.1. Main Findings

In this nationwide patient-level study of 69,349 adults hospitalized with chronic ulcers, malignancy recorded among secondary diagnoses was associated with higher odds of in-hospital mortality after adjustment for available demographic and hospitalization-level variables. This association persisted in a sensitivity model additionally adjusted for the number of non-malignant secondary diagnoses recorded during the analytical hospitalization; the near-identical estimates in Model 2 and Model 3 support the robustness of the main association after accounting for overall coded non-malignant comorbidity burden.

The association was most evident in the overall cohort and among single-admission patients. In recurrent patients, the point estimates were above 1, but confidence intervals were wide and the association did not reach statistical significance. Because only eight deaths occurred among recurrent patients with C00–C97 codes, this subgroup analysis should be interpreted as exploratory and underpowered rather than as evidence of absence of association.

Secondary analyses suggested clinical heterogeneity within the broad C00–C97 exposure. The strongest crude associations with mortality were observed for metastatic or secondary malignant neoplasm codes, malignant neoplasm without specified site, and solid non-skin malignancies. In contrast, skin malignancy codes showed low observed in-hospital mortality and were not interpreted as protective because this category is clinically heterogeneous and includes many non-melanoma skin cancers.

### 4.2. Clinical Interpretation of Coded Malignancy

The observed association between malignancy recorded among secondary diagnoses and in-hospital mortality is clinically plausible. In patients hospitalized with chronic ulcers, death is unlikely to reflect the wound alone. It more likely reflects the combined influence of ulcer phenotype, acute illness, multimorbidity, and reduced physiological reserve. These patients are often older and may also have cardiovascular disease, diabetes, renal impairment, infection, immobility, malnutrition, or other conditions that worsen inpatient outcomes.

Within this context, coded malignancy may identify an additional high-risk clinical profile. Cancer can influence prognosis through inflammation, immune dysfunction, cachexia, nutritional deterioration, treatment-related toxicity, increased susceptibility to infection, and reduced functional reserve. However, the present administrative data cannot determine whether cancer was active or advanced, whether it directly contributed to death, or whether the code reflected broader clinical deterioration. Therefore, C00–C97 codes recorded among secondary diagnoses should be interpreted as an administrative indicator of increased inpatient risk and limited physiological reserve, rather than as evidence of a direct causal effect on mortality.

Clinically, malignancy codes recorded among secondary diagnoses in hospitalized chronic ulcer patients may help identify individuals who could benefit from earlier multidisciplinary assessment.

The supportive internal consistency analysis strengthened the plausibility of malignancy coding in this dataset. Some patients with C00–C97 codes also had additional codes compatible with oncological burden or cancer-related care, including metastatic or unspecified malignant neoplasm codes, cachexia, malnutrition, anemia in neoplastic disease, chemotherapy or radiotherapy-related care codes, and personal history of malignancy. Because linkage with cancer registries or clinical records was not available, these findings indicate internal coding coherence rather than diagnostic confirmation.

### 4.3. Interpretation of Single-Admission and Recurrent-Patient Analyses

The stronger association observed among single-admission patients may partly reflect deaths occurring during an early admission, before recurrent hospitalizations could occur. This interpretation is clinically plausible but requires caution, because admission pattern depends on survival time. Patients who die early cannot later be classified as recurrent, which may distort comparisons between single-admission and recurrent groups.

For recurrent patients, the index-episode approach was a pragmatic patient-level strategy. The death admission was selected for patients who died, whereas the last observed admission was selected for survivors. This choice captures clinically relevant records, but it also means that recurrent-patient comparisons are partly shaped by how long patients survived during the study period. This may help explain why estimates differed between single-admission and recurrent patients.

For these reasons, the single-admission and recurrent-patient analyses were considered secondary and exploratory. Recurrent-patient estimates were imprecise, based on only eight exposed deaths, and should not be interpreted as evidence that no association exists. Confirmation in datasets with longer follow-up, cancer-stage information, outpatient care data, and clinical wound-severity measures is needed.

### 4.4. Implications for Clinical Care and Health-System Planning

From a clinical perspective, malignancy recorded among secondary diagnoses may help identify hospitalized chronic ulcer patients at higher risk of in-hospital death. The finding is particularly relevant for wound-care teams because chronic ulcers can function as visible markers of systemic decline, not only as local tissue defects. In patients with both chronic ulcers and malignancy codes, clinicians should consider broader assessment of nutritional status, infection risk, functional decline, systemic inflammatory burden, and possible advanced or metastatic disease.

These findings support integrated care pathways involving dermatology, oncology, surgery, internal medicine, infectious disease specialists, nutrition support, and, when appropriate, palliative care teams. The high mortality observed among patients with pressure ulcers and arterial ulcers who also had malignancy codes suggests that the combination of tissue vulnerability, impaired perfusion or pressure-related injury, and systemic oncological burden may identify particularly fragile inpatients. However, these ulcer-category findings are descriptive and should not be interpreted as formal effect modification without additional interaction analyses.

From an administrative and public health perspective, the study illustrates the value of national hospitalization databases for identifying high-risk subgroups among patients with chronic ulcer disease. Although administrative data lack clinical granularity, they allow large-scale assessment of objective outcomes such as in-hospital death and may support risk stratification, resource planning, and the development of integrated care models for complex patients.

### 4.5. Strengths and Limitations

This study has several strengths. It was based on a nationwide administrative hospitalization database covering Romanian public hospitals over a six-year period and included a large cohort of 69,349 adult patients hospitalized with chronic ulcers. The analysis was performed at patient level, reducing over-representation of patients with repeated admissions, and used an objective outcome recorded in discharge data: in-hospital death. The analytical cohort was explicitly defined using an index-episode strategy, summarized in a flow diagram, and assessed through crude, adjusted, and sensitivity models. Additional analyses examined internal coding consistency, malignancy subcategories, and rare-event modeling in the recurrent subgroup.

Several limitations should also be acknowledged. First, the study relied on administrative hospitalization data, which depend on coding accuracy and completeness. Misclassification, undercoding, and differences in coding practices across hospitals may have affected the identification of both chronic ulcers and malignancy. Data quality checks and RECORD/STROBE-oriented reporting improved transparency, but they cannot replace validation against medical records or cancer registry data.

Second, several clinically important variables were unavailable, including wound size, wound duration, infection severity, microbiology, laboratory parameters, nutritional status, functional status, cancer stage, cancer treatment status, performance status, and cause of death. Therefore, we could not determine whether deaths were directly related to malignancy, ulcer complications, infection, vascular disease, or other acute conditions. Residual confounding by cancer severity, treatment-related factors, clinical severity, acute illness, comorbidity burden, frailty, nutritional status, and functional impairment cannot be excluded.

Third, malignancy was identified using ICD-10 C00–C97 codes recorded among secondary diagnoses. Although supportive oncological codes were examined for internal consistency, the database could not reliably distinguish active cancer from treated cancer, cancer in remission, historical cancer, metastatic disease not explicitly coded as such, or treatment-related oncological status. The exposure should therefore be interpreted as administratively coded malignancy rather than clinically verified active cancer.

Fourth, the number of non-malignant secondary diagnoses was used only as a pragmatic administrative proxy for coded comorbidity burden. It is not equivalent to validated comorbidity indices such as Charlson or Elixhauser. Such indices were not constructed because their malignancy-related components overlap with the primary exposure definition, and the available data did not allow reliable separation of chronic comorbidities from acute complications or severity-related coding. In addition, the number of coded secondary diagnoses may reflect coding intensity, documentation completeness, hospital practices, reimbursement-related coding behavior, and episode complexity, not only true multimorbidity.

Fifth, stratification by single versus multiple hospitalizations is susceptible to survival-related distortion, because death during an early admission prevents later classification as a recurrent patient. Recurrent-patient models were also underpowered, with only eight exposed deaths. These analyses should therefore be interpreted as exploratory and hypothesis-generating.

Finally, because of the retrospective observational design, the results demonstrate association rather than causation. Malignancy recorded among secondary diagnoses should be interpreted as a marker associated with increased inpatient mortality risk, not as evidence of a direct causal effect.

### 4.6. Future Research

The consistently higher observed mortality among patients with pressure ulcers and arterial ulcers who also had C00–C97 codes suggests that the combination of malignancy-related systemic vulnerability with pressure-related tissue injury or impaired arterial perfusion may identify a particularly fragile inpatient phenotype. However, this finding was descriptive and should be tested in future studies using formal interaction analyses between malignancy status and chronic ulcer category.

Future studies should validate these findings using clinically enriched datasets that include wound characteristics, cancer stage, treatment status, laboratory parameters, nutritional indicators, infection severity, functional status, and post-discharge outcomes. Linkage between hospitalization databases, cancer registries, outpatient wound-care data, and mortality registries would be particularly valuable for distinguishing active malignancy from previous cancer history and for evaluating the role of cancer stage and metastatic disease.

Further research should also assess whether malignancy improves prognostic models for hospitalized chronic ulcer patients beyond age, ulcer category, admission type, and comorbidity burden. Prospective studies could clarify the mechanisms linking malignancy, impaired wound healing, systemic vulnerability, and in-hospital death, while also identifying clinical pathways for earlier risk recognition and integrated management.

## 5. Conclusions

Malignancy recorded among secondary diagnoses was associated with higher odds of in-hospital mortality among patients hospitalized with chronic ulcers after adjustment for demographic and hospitalization-level variables. This association persisted in a sensitivity model accounting for the number of non-malignant secondary diagnoses. It was most evident in the overall cohort and among single-admission patients, whereas recurrent-patient estimates were imprecise and should be interpreted cautiously.

Exploratory analyses suggested that higher mortality was mainly observed among patients with metastatic or secondary malignant neoplasm codes, malignant neoplasm without specified site, and solid non-skin malignancies. Coded malignancy may therefore serve as a pragmatic administrative marker of increased inpatient risk and may support broader risk stratification and integrated care approaches. These findings should not be interpreted as evidence that malignancy directly causes in-hospital mortality, because the observational design, administrative exposure definition, and lack of cancer-stage information preclude causal inference. Nevertheless, they provide clinically useful opportunities to enhance awareness, improve risk identification, and guide more proactive and coordinated management strategies for hospitalized patients with chronic ulcers.

## Figures and Tables

**Table 1:** Baseline characteristics of patients according to malignancy recorded among secondary diagnoses in the index-episode cohort.

Variable	Category	No Malignancy *n* (%)	Malignancy C00–C97 *n* (%)	*p*-Value
Age, years	Mean (SD)	66.2 (13.8)	70.7 (11.5)	<0.001
Age group	<45 years	4810 (7.1)	53 (2.9)	<0.001
	45–54 years	8223 (12.2)	112 (6.1)	
	55–64 years	14,677 (21.7)	314 (17.1)	
	65–74 years	19,556 (29.0)	591 (32.2)	
	75–84 years	15,589 (23.1)	600 (32.7)	
	≥85 years	4657 (6.9)	167 (9.1)	
Sex	Female	32,634 (48.3)	883 (48.1)	0.837
	Male	34,878 (51.7)	954 (51.9)	
Area of residence	Urban	33,219 (49.2)	1052 (57.3)	<0.001
	Rural	34,293 (50.8)	785 (42.7)	
Type of admission	Non-emergency/other	46,869 (69.4)	1331 (72.5)	0.006
	Emergency	20,643 (30.6)	506 (27.5)	
Chronic ulcer category	Venous ulcer (I83.x)	30,221 (44.8)	423 (23.0)	<0.001
	Arterial ulcer (I70.23)	8082 (12.0)	169 (9.2)	
	Diabetic ulcer (E1x.73)	3851 (5.7)	43 (2.3)	
	Pressure ulcer (L89)	4554 (6.7)	223 (12.1)	
	Non-classified lower-limb ulcer (L97)	12,383 (18.3)	323 (17.6)	
	Chronic skin ulcer NEC (L98.4)	8421 (12.5)	656 (35.7)	
Hospitalization pattern	Single	49,101 (72.7)	1392 (75.8)	0.004
	Multiple	18,411 (27.3)	445 (24.2)	
In-hospital outcome	Survived	66,307 (98.2)	1764 (96.0)	<0.001
	Died in hospital	1205 (1.8)	73 (4.0)	

Note: Values are presented as *n* (%) unless otherwise specified. Malignancy exposure was defined as the presence of at least one ICD-10 C00–C97 code recorded among secondary diagnoses in the analytical hospitalization. For recurrent patients, this was the index hospitalization. Group comparisons were performed using the χ^2^ test for categorical variables and the *t*-test or Mann–Whitney U test for continuous variables, as appropriate. *p*-values refer to comparisons between patients with and without ICD-10 C00–C97 codes recorded among secondary diagnoses.

**Table 2:** Crude association between malignancy recorded among secondary diagnoses and in-hospital mortality.

Cohort	Overall	Single	Multiple (Index Episode)
*N*	69,349	50,493	18,856
C00–C97 *n*	1837	1392	445
Deaths in C00–C97	73	65	8
Mortality C00–C97	3.97%	4.67%	1.80%
Mortality without C00–C97	1.78%	1.98%	1.28%
Crude OR	2.28	2.43	1.42
95% CI	1.79–2.90	1.88–3.14	0.70–2.88
χ^2^ *p*-value	<0.001	<0.001	0.335
Fisher’s exact *p*-value	<0.001	<0.001	0.289

Note: Odds ratios compare the odds of in-hospital death among patients with at least one ICD-10 C00–C97 code recorded among secondary diagnoses versus those without such codes. χ^2^ *p*-values are reported for all comparisons; Fisher’s exact *p*-values are additionally reported to account for sparse event counts, particularly in the recurrent/index-episode subgroup. CI: confidence interval; OR: odds ratio.

**Table 3:** Adjusted logistic regression models for the association between malignancy recorded among secondary diagnoses and in-hospital mortality.

Cohort	Model	Adjustment Set	*N* Included in Model	aOR for C00–C97	95% CI	*p*-Value
Overall	M1	Age group + sex + admission type	69,349	2.42	1.88–3.10	<0.001
Overall	M2	M1 + ulcer category + hospitalization pattern	69,349	1.87	1.42–2.45	<0.001
Overall	M3 sensitivity	M2 + non-malignant diagnosis count	69,349	1.88	1.42–2.47	<0.001
Single	M1	Age group + sex + admission type	50,493	2.50	1.91–3.27	<0.001
Single	M2	M1 + ulcer category	50,493	1.93	1.44–2.58	<0.001
Multiple/index	M1	Age group + sex + admission type	18,856	1.73	0.84–3.59	0.137
Multiple/index	M2	M1 + ulcer category	18,856	1.51	0.70–3.25	0.293
Multiple/index	Firth sensitivity	Penalized M2	18,856	1.58	0.75–3.33	0.226

Note: M1 was adjusted for age group, sex, and type of admission. M2 was additionally adjusted for chronic ulcer category and hospitalization pattern in the overall cohort. In stratified analyses, hospitalization pattern was not included because patients were already stratified as single-admission or recurrent. M3 was a sensitivity model additionally adjusted for the number of non-malignant secondary diagnoses recorded during the analytical hospitalization. Firth sensitivity refers to Firth penalized logistic regression in the recurrent subgroup because of the small number of exposed deaths. No missing values were identified for the variables included in the adjusted models; therefore, complete-case analysis did not exclude any patients from the regression models.

## Data Availability

The data underlying this study were obtained from the National Institute of Public Health under a data-sharing agreement and cannot be publicly shared by the authors. Aggregated results, data quality checks, model specifications, and supplementary analyses are provided in the Supplementary Materials.

## Supplementary Materials

The following supporting information can be downloaded at: https://www.mdpi.com/article/10.3390/jcm15135261/s1, Table S1: Data quality checks, missing-data assessment, model-specific analytical sample sizes, distribution of the non-malignant secondary diagnosis count, sensitivity models using this count as a comorbidity-burden proxy, Firth penalized recurrent-subgroup sensitivity analysis, STROBE/RECORD reporting alignment, and model fit and apparent in-sample performance metrics; Table S2: Non-mutually exclusive malignancy categories, solid non-skin malignancy subcategories, and distribution of malignant codes/categories per patient; Table S3: Individual malignant ICD-10 codes recorded among secondary diagnoses and explored in relation to in-hospital mortality, including the complete code list and descriptive code-level associations; Table S4: Supportive internal consistency analysis of oncological diagnostic patterns and cancer-related care codes; Table S5: Descriptive in-hospital mortality among patients with C00–C97 codes by chronic ulcer category.
